# Mechanism of stress-driven composition evolution during hetero-epitaxy in a ternary AlGaN system

**DOI:** 10.1038/srep25124

**Published:** 2016-04-26

**Authors:** Chenguang He, Zhixin Qin, Fujun Xu, Lisheng Zhang, Jiaming Wang, Mengjun Hou, Shan Zhang, Xinqiang Wang, Weikun Ge, Bo Shen

**Affiliations:** 1 State Key Laboratory of Artificial Microstructure and Mesoscopic Physics, School of Physics, Peking University, Beijing 100871, China; 2 Collaboration Innovation Center of Quantum Matter, Beijing 100084, China

## Abstract

Two AlGaN samples with different strain were designed to investigate mechanism of stress-driven composition evolution. It is discovered that AlGaN grown on AlN or (AlN/GaN superlattices (SLs))/GaN both consist of two distinct regions with different compositions: transition region and uniform region, which is attributed to the compositional pulling effect. The formation of the transition region is due to the partial stress release caused by the generation of misfit dislocations near the hetero-interface. And the Al composition in the uniform region depends on the magnitude of residual strain. The difference in relaxation degree is 80.5% for the AlGaN epilayers grown on different underlayers, leading to a large Al composition difference of 22%. The evolutionary process of Al composition along [0001] direction was investigated in detail.

In the past decades, AlInGaN alloys have been widely applied as cladding layers and active regions in light-emitting diodes (LEDs), laser diodes (LDs), and photodetectors (PDs)^1^,^2^,^3^. Advances in AlInGaN also show great application prospects in high electron mobility transistors (HEMTs)^4^. Undoubtedly, an accurate control of composition is a prerequisite to fabricate these devices. Because the variation of alloy composition has a significant influence on the performance of the above devices^5^,^6^. Unfortunately, it remains a challenge for metalorganic vapor phase epitaxy (MOVPE) to accurately control alloy composition, which is usually affected by growth conditions and reactor structures.

Besides, the compositional pulling effect (CPE) caused by mismatch stress is also an important factor that cannot be ignored^7^,^8^,^9^,^10^,^11^,^12^. It was reported that AlGaN barriers grown on different buffers had different Al compositions and growth rates in AlGaN/GaN heterostructures^7^. Another group found that the 2DEG carrier concentration increased with the thickness of AlInGaN barrier due to the CPE^11^. It demonstrates that CPE have an obvious effect on the incorporation of Al, In, and Ga adatoms. The above quoted studies provide a useful guidance for the control of performance parameters in HEMTs. However, in consideration of the applications in LEDs, LDs, and PDs, the composition evolutionary process in thick AlGaN epilayer needs to be investigated in detail. Although the gradient Al composition variation in the whole range of 1.7 μm has been reported for AlGaN grown on thin-GaN-coated sapphire, the structure is not common for device applications^8^. In actual device structures, AlN and GaN underlayers are usually grown to a-few-micron-thick for improving crystal quality and releasing stress^1^,^2^,^3^. In this situation, the stress state in AlGaN epilayers will be quite different. Therefore, it is of great significance to investigate the composition evolutionary process along the growth direction in such a system.

In this paper, two AlGaN samples with different strain were designed to investigate stress-driven composition evolution. X-ray diffraction reciprocal space mappings (XRD RSMs) were used to characterize the composition and strain in the AlGaN epilayers. Energy dispersive spectroscopy (EDS) were applied to provide high-accuracy and high-space-resolution Al composition information. Transmission electron microscope (TEM) measurements were performed to examine the evolution of dislocations. And then, the Al composition evolution processes along [0001] direction were analyzed in detail.

As shown in Fig. 1(a,b), two samples with different strain were prepared for the experiments. The only difference between sample A and B lies in the underlayers, and the growth conditions are the same for the AlGaN epilayers. The details about the samples can be found in the section of sample preparation. First, sample A and B were characterized by XRD RSMs. To facilitate the analysis, the XRD RSMs for sample A and B were put together for comparison, as shown in Fig. 2(a). Diffraction peaks coming from AlN template, AlGaN in sample A, and AlGaN in sample B, as well as GaN template can be well identified. As designed, sample A and B exhibit large strain difference in AlGaN epilayers by using different underlayers. The AlGaN epilayer in sample A has a relaxed degree of 19.5%, which means AlGaN epilayer bears strong compressive stress^13^. In the case of sample B, however, the strain in AlGaN epilayer has been completely relaxed by AlN/GaN SLs. In the meantime, an Al composition difference of 21.3% between sample A and B can be clearly observed. Considering that AlGaN epilayers in sample A and B were prepared under the same growth conditions, it is reasonable to believe that the large Al composition difference results from the large strain discrepancy between sample A and B.

Figure 2(b) shows the Al composition profiles of sample A and B along [0001] direction, which were measured by TEM EDS line scans. Obviously, for AlGaN epilayers grown on AlN and (AlN/GaN SLs)/GaN underlayers, both of them consist of two distinct regions with different compositions: transition region (grey region) and uniform region. In the transition region, the Al composition varies sharply in a small range. While in the uniform region, the Al composition remains nearly the same for several hundred nanometers. For AlGaN epilayers grown on different underlayers, there exists an Al composition difference of 22% in the uniform region. It can be seen that the Al composition difference ∆ measured by TEM line scans is nearly equivalent to that measured by XRD RSMs. Considering that the transition region only occupies a small part in AlGaN epilayers, the residual strain determined by the XRD RSMs can be approximately equal to the residual strain in the uniform region. Quantitatively, for AlGaN epilayers grown on different underlayers, a large difference of 80.5% in relaxation degree leads to a large Al composition difference of 22% in the uniform region.

As for the transition region, in view of the limited space-resolution of TEM EDS line scan, the Al profile details along [0001] direction cannot be clearly displayed. To solve this problem, cross-sectional TEM EDS mappings were further performed on sample A and B. Figure 3(a,b) show the cross-sectional morphology and qualitative distribution of Al, Ga, and N elements in the selected area (green frame) for sample A and B, respectively. The corresponding Al composition profiles along the red arrows are shown in Fig. 3(c,d), respectively. Evidently, more details are revealed benefiting from a fine scanning step of TEM EDS mappings, which is on the scale of 1 nm. For sample A, the Al composition falls rapidly in the initial stage of AlGaN growth, which is corresponding to the transition region (0–15 nm). When the thickness exceeds 15 nm, the Al composition enters into the uniform region with an Al composition of 58%. In the case of sample B, the evolutionary process seems to be complicated due to the existence of AlN/GaN SLs. It is found that superlattice structure is gradually formed. For the lower part of the nominal AlN/GaN superlattice structure, the interfaces between barriers and wells are difficult to identify from the cross-sectional morphology in Fig. 3(b). Simultaneously, no Al composition oscillation can be found from 0 nm to 30 nm in Fig. 3(d). On the contrary, a monotonously increasing Al composition is presented. When the thickness exceeds 30 nm, the oscillation of Al composition becomes more and more apparent. Finally, the Al composition in the nominal AlN/GaN SLs oscillates within a relatively stable range. For the subsequent AlGaN epilayer, it exhibits a similar variation trend with that in sample A. The Al composition falls rapidly in the range of 52–65 nm (transition region), and then enters into the uniform region with an Al composition of 38%.

These phenomena can be well explained by the CPE. For sample A, at the very begining of the transition region, the compressive stress caused by lattice mismatch pulls Ga adatoms from the AlGaN epilayer to reduce the strain energy, resulting in the deposition of AlGaN containing high Al composition^12^. As the growth in the transition region continues, partial compressive stress is rapidly released due to the generation of misfit dislocations near the AlGaN/AlN interface^14^. As a result, more and more Ga adatoms are incorporated into the AlGaN epilayer, leading to an obvious decrease of the Al composition along [0001] direction. After the rapid stress release near the AlGaN/AlN interface, the residual mismatch strain will be relaxed slowly in the subsequent epitaxy. In such circumstances, the degree of CPE keeps relatively stable. As a result, a nearly constant Al composition along [0001] direction can be observed in the uniform reigon.

Figure 4(a,b) show the cross-sectional bright-field TEM images for sample A at the same area, under two-beam conditions with *g* = [0002] and *g* = [11−20], respectively. Based on the standard Burgers vector analysis using the invisibility criterion *g·b* = 0, the dislocation type can be identified. Screw TDs are visible with *g* = [0002], while edge TDs are visible with *g* = <11−20>. It can be seen that a few dislocations are generated at the AlGaN/AlN interface and propagate vertically along the growth direction, which confirms that mismatch stress is partly released near the interface by the generation of misfit dislocations^14^,^15^. In addition, it should be noted that some pre-existing edge dislocations bend away from [0001] direction, which is also a strain relaxation channel in AlGaN epilayer bearing compressive stress^16^,^17^. The average value of the inclined angles is about 20°. The x-ray rocking curve values of (002) and (102) plane for the AlN template are 151 and 508 arcsec, respectively. The corresponding edge dislocation density is about 3 × 10^9 ^cm^−2^. Based on these, it can be calculated that average strain relaxation caused by the incline of edge dislocations is 0.0005946 assuming that all pre-existing edge dislocations have inclined^16^. For Al_0.58_Ga_0.42_N in sample A, the value of total strain relaxation is estimated to be 0.001708 by XRD RSMs. In other words, the strain relaxation caused by the incline of edge dislocation account for 35% at most in the total strain relaxation. Simultaneously, considering that the stress relaxation caused by the incline of edge dislocations is a slow process. Therefore, it is believed that the inclined dislocations has a weak effect on the Al composition evolution in the uniform region.

In the case of sample B, in the initial stage of AlN/GaN SLs growth, the Al adatoms are difficult to be incorporated into the epilayer because of the large tensile stress coming from GaN template. As the growth continues, tensile stress is dramaticly released due to the generation of misfit dislocations and cracks in the AlN/GaN SLs^15^,^18^,^19^,^20^,^21^, as shown in Fig. 4(c–f). In consequence, more and more Al adatoms were incorporated into the epilayer, resulting in the formation of a gradient-Al-composition AlGaN epilayer, which accounts for the missing superlattice interfaces. When the Al composition ramps to a value that is close to the equivalent Al composition of AlN/GaN (2 nm/1 nm), the mismatch stress doesn’t change much. After that, the superlattice interfaces can be gradually identified. Finally, the Al composition oscillates within a relatively stable range. As for the subsequent AlGaN epilayer, it bears a compressive stress coming from the nominal AlN/GaN SLs in the area far from the cracks. The Al composition varies rapidly, accompanying with the generation of misfit dislocations, as shown in Fig. 4(c,d). As the growth processed, the whole AlGaN epilayer relaxed by the combined action of misfit dislocations and cracks. The detailed mechanism of stress relaxation can be found in our previous work^19^. Then it can be well understood why the AlGaN epilayer in sample B shows a similar composition evolutionary process with that in sample A. The difference lies in that the residual mismatch stress in the uniform region, which results in a large Al composition difference of 20% (close to the values mentioned above).

In summary, it is discovered that AlGaN epilayer grown on a foreign underlayer consists of a compositional transition region and a compositional uniform region, which is attributed to the compositional pulling effect (CPE). The formation of transition region is due to the partial stress release caused by the generation of misfit dislocations near the hetero-interface. And the Al composition in the uniform region depends on the magnitude of residual strain. In addition, in the initial stage of SL growth, the Al compositions in barriers and wells tend to be consistent due to the strong CPE, resulting in an indistinct interface. For AlGaN grown on AlN and ((AlN/GaN SLs)/GaN) underlayers, the difference in relaxation degree is 80.5% for AlGaN epilayers grown on different underlayers, leading to a large Al composition difference of 22%. It implies that the CPE plays a very important role in the hetero-epitaxy of AlGaN.

## Methods

### Sample Preparation

**Figure 1:**
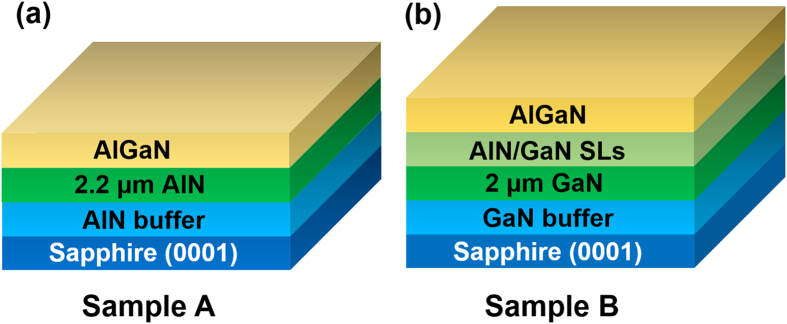
(**a**) Structure schematic illustration for sample A. The deposition was initiated from an AlN buffer layer grown on (0001) sapphire substrate, followed by a 2.2-μm-thick AlN layer and a 625-nm-thick AlGaN layer. (**b**) Structure schematic illustration for sample B. The sample structure consists of a GaN buffer layer grown on (0001) sapphire substrate, a 2-μm-thick GaN layer, a 20-period AlN/GaN (2 nm/1 nm) superlattice (SL) layer, and a 600-nm-thick AlGaN layer.

The samples were grown on 2-in. (0001) sapphire substrates by low pressure metal organic vapor phase epitaxy (LP-MOVPE), using an Aixtron vertical close-coupled showerhead (CCS) system. Trimethylaluminium (TMAl), Trimethylgallium (TMGa), and ammonia (NH3) were utilized as Al, Ga, and N sources, respectively. The cross section schematic of the AlGaN samples is shown in Fig. 1(a,b). For sample A, the deposition was initiated from an AlN buffer layer grown on (0001) sapphire substrate, followed by a 2.2-μm-thick AlN layer and a 625-nm-thick AlGaN layer. The crystal quality of AlN template was improved by a 7-second-nitridation pretreatment on sapphire based on our previous work^22^. While for sample B, the sample structure consists of a GaN buffer layer grown on (0001) sapphire substrate, a 2-μm-thick GaN layer, a 20-period AlN/GaN (2 nm/1 nm) superlattice (SL) layer, and a 600-nm-thick AlGaN layer. The details about (AlN/GaN SLs)/GaN can also be found in our previous work^19^. The AlGaN epilayers were grown at 1170 °C under H_2_ ambient with total III flow rate of 50 μmol/min (TMAl flow/(TMAl flow + TMGa flow) = 5.7%). The reactor pressure and V/III ratio during AlGaN growth process were nominally 100 mbar and 1064, respectively. The growth rates of AlGaN epilayers in sample A and B are 0.132 μm/h and 0.176 μm/h, respectively. The thickness and temperature were monitored *in situ* using LayTec EpiTT optical (405 nm and 950 nm) reflectance.

### Measurements

RSMs of these samples were taken around the asymmetric (−105) plane, using a Bruker AXS D8 Discover HRXRD. The TEM-ready samples were prepared using the *in situ* FIB lift out technique on an FEI Dual Beam FIB/SEM. Structural characterizations were realized by a FEI Tecnai Osiris TF-20 FEG/TEM equipped with EDS.

## Additional Information

**How to cite this article**: He, C. *et al.* Mechanism of stress-driven composition evolution during hetero-epitaxy in a ternary AlGaN system. *Sci. Rep.* **6**, 25124; doi: 10.1038/srep25124 (2016).

## Figures and Tables

**Figure 2:**
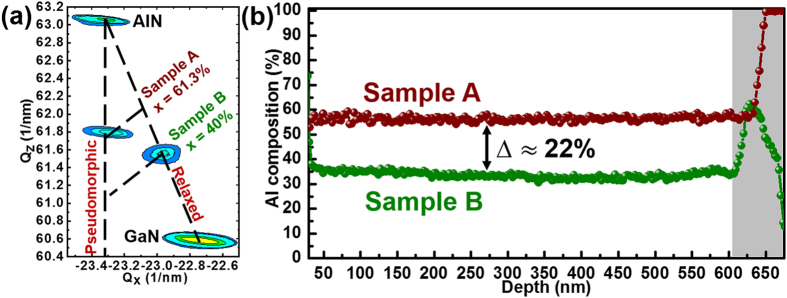
(**a**) XRD RSMs of the asymmetric (−105) plane for sample A and B. Sample A has a relaxation degree of 19.5% and an Al composition of 61.3%. Sample B has a relaxation degree of 100% and an Al composition of 40%. (**b**) The Al composition profiles along [0001] direction for sample A and B. There exists an Al composition difference of 22% in the uniform region (about 0–600 nm). The grey area is corresponding to the transition region (>600 nm).

**Figure 3:**
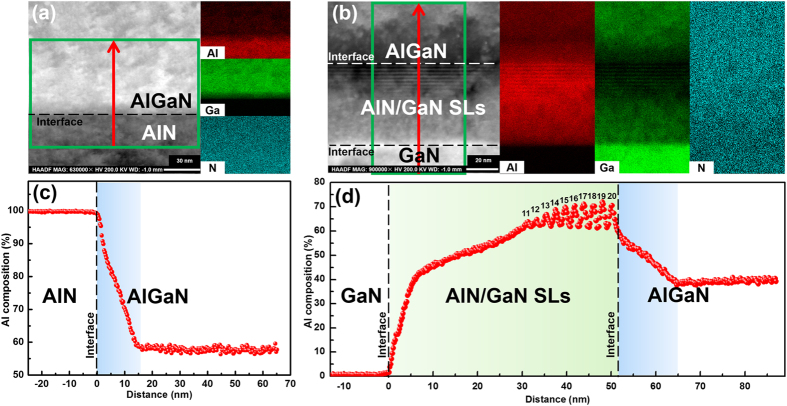
Cross-sectional TEM EDS mappings for (**a**) sample A and (**b**) B with a high space-resolution of 1 nm. The Al composition profiles along the red arrows ([0001] direction) for (**c**) sample A and (**d**) B, respectively.

**Figure 4:**
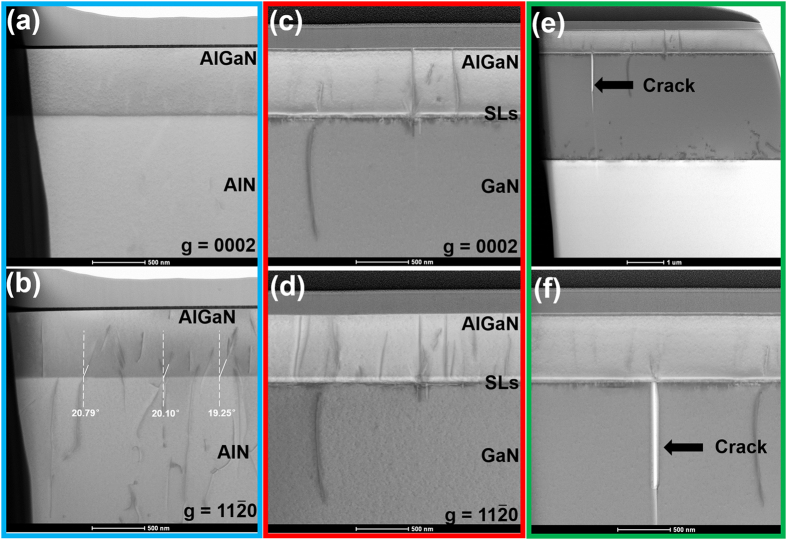
Cross-sectional TEM images for sample A with (**a**) g = [0001] and (**b**) g = [11, 12, 13, 14, 15, 16, 17, 18, 19, 20]. A few dislocations are generated near the AlGaN/AlN interface. It can also be seen that some pre-existing edge dislocations bend away from [0001] direction by an average angle of 20°. Cross-sectional TEM images for sample B with (**c**) g = [0001] and (**d**) g = [11, 12, 13, 14, 15, 16, 17, 18, 19, 20]. Many dislocations are generated near the (AlN/GaN SLs)/GaN interface. In addition, a few dislocations are generated near the AlGaN/(AlN/GaN SLs) interface. Crack generated in sample B during epitaxy are shown in (**e**,**f**).
